# Stages in Theory and Experiment. Fuzzy-Structuralism and Piagetian Stages

**DOI:** 10.1007/s12124-022-09702-7

**Published:** 2022-06-15

**Authors:** Mark A. Winstanley

**Affiliations:** Friedrich-Schiller-Universitaet, Ernst-Haeckel-Haus, Berggasse 7, D-07745 Jena, Germany

**Keywords:** Stages of Cognitive Development, Situated cognition, Crisis of Variability, Fuzzy structuralism, Fuzzy stages

## Abstract

The theory of stages in cognitive development is one of Jean Piaget’s enduring legacies, but it has also borne the brunt of much criticism. It maintains that intelligence develops in an invariant sequence of stages, and, in this paper, I situate Piaget’s conceptions of stages historically and functionally in the context of genetic epistemology, his research programme. I highlight some of the objections raised, and I show how the disparity between the conceptions of theoretical and empirical stages in Piaget’s theory is commensurate with the fuzzy-structuralist model of the relationship between theory and empirical research conceived by Rudolf Seising on the basis of Lofti A. Zadeh’s fuzzy set theory. Further, I propose a fuzzy conception of the notion ‘stage’, which not only captures its ordinary use in fuzzy space between theory and empirical research but also does justice to both the construct validity and quantitative variability of stages in empirical research. I therefore open a fuzzy-structuralist perspective on the Crisis of Variability afflicting Piaget’s stage theory during the 1970s and conclude retrospectively that the rift it caused was not necessary since the invariance and variability of stages is not irreconcilable.

## Stages of Cognitive Development

It is testimony to Jean Piaget’s legacy that people still associate stages with the cognitive development of children even in non-academic circles. Endurance and recognition in the general public are, however, poor measures of the acceptance Piaget’s stage theory of cognitive development enjoys amongst developmental psychologists. Kesselring (2009, 375) makes a catalogue of nine objections against Piaget’s stage theory. Last but not least in the list is the coherence of the theory itself.

The stage theory is used in Piaget’s work in two fundamentally different ways. As a rule, three stages are discerned in experimental contexts. Children are set specific tasks designed to reveal cognitive abilities, and their performance is usually divided into success, partial success or failure. Besides categorising task-related performance according to degrees of success, Piaget also used ‘stage’ in his stage theory to designate characteristic forms of cognitive behaviour that manifest in an invariant sequence during cognitive development. In contrast to the consistency in the number of stages in experimental contexts, the number of stages in the latter, theoretical, context varies considerably (Kesselring, 2009, 379). Some of the variety has its roots in Piaget’s own conceptual development. However, even after making allowances for the conceptual development of his stage theory, inconsistencies in the number of stages still remain in Piaget’s work (Kesselring, 2009, 379).

### Development of Piaget’s Stage Theory

1936 is a watershed in the development of Piaget’s stage theory. From 1923 to 1929, he advocated a three-stage theory of cognitive development. In the first stage, termed *autism*, the child’s thinking was supposed to be adualistic and non-directed; Piaget thought it lasted until the third year and was succeeded by egocentrism. In the second, the *egocentric* stage, the child was not yet supposed to be able to distinguish clearly between his/her own perspective and the perspectives of others. At the final stage, beginning at about the age of seven, Piaget thought that deductive reasoning and social reciprocity characterised the child’s thinking. According to this early model of cognitive development, progression through the stages was caused by *decentration*, the process of overcoming natural biases inherent in cognitive activities. However, motivated by meticulous observations of his own children, Piaget had abandoned the three-stage model of these early years by 1936 at the latest. He replaced autism with a stage of practical intelligence lasting ca 1½ years, the *sensorimotor* stage, and distinguished the decentration processes involved in this stage from those combating egocentrism in the later stages (Kesselring, 2009, 380) .

Since Piaget did not advocate his early stage model—autism, egocentrism, deductive thinking and social reciprocity—after 1936, it can be eliminated from any enumeration of valid stage models. Nevertheless, accommodating observations did not eliminate variety in the stage theories advocated by Piaget altogether. Post 1936, Piaget still did not consistently refer to a single stage model. Although three and four-stage models prevail, there are a few instances of five and six-stage models in Piaget’s works. In addition, Piaget does not always consistently refer to one of the prevailing models in one-and-the-same work but often uses a mixture. Not surprisingly opinion amongst Piaget scholars is equally divided, some advocating three stages, whilst others prefer four. The stages of cognitive development according to the three-stage theory are sensorimotor, concrete-operational and formal-operational; for the four-stage theory, in contrast, they are sensorimotor, preoperational, concrete-operational, and formal-operational (Kesselring, 2009, 380–1). Moreover, decentration appears to cease its operation by the concrete-operational stage in the four-stage model but continues to operate throughout the preoperational and concrete-operational phases, in the three-stage variant.

### Characteristics of the Stages of Cognitive Development

Despite differences in the number of stages, the models have many features in common. In particular, stages are characterised by specific cognitive structures; for instance, the group of displacements giving rise to object permanence at the sensorimotor stage; natural numbers, the eight groupings regulating the manipulation of discrete objects as well as the eight corresponding infra-logical groupings for spatio-temporal relations at the concrete-operational stage; and the single grouping formed by interpropositional operations of thought at the formal-operational stage, which combines the separate groupings of the concrete-operational stage (Piaget, 2001, 46ff; see also Piaget and Grize 1972, 87ff). The stages also follow each other in an invariant sequence in the models. Although progression through the stages may be accelerated or retarded, it is not possible to skip intermediate stages in the sequence since each stage is a prerequisite for subsequent stages.

The transition from one stage to the next gives rise to integration and consolidation at the stage attained. Integration is due to a conservative tendency in cognitive development, which preserves the structures already attained at a previous stage in the structures of subsequent stages. Consolidation, on the other hand, has two aspects: preparation and completion. Each stage prepares the ground for its successor whilst the structures characterising each stage represent the culmination of the preparation that took place at previous stages. Finally, from a dynamic point of view the structures at any stage are forms of relative equilibrium, and equilibration is the driving force which transforms a state of cognitive development in relative disequilibrium into a new state in equilibrium (Piaget, 1977, 815–6; see also Brainerd 2000; Inhelder, 2000).

Equilibration is, according to Piaget, one of the principal mechanisms mediating the growth of knowledge. However, explanatory primacy in science rarely reflects the actual order of discovery, the most fundamental causes often being the last to come to light because the deepest phenomenal penetration is required. Genetic epistemology is no exception to this rule; its research culminated in the investigation of the developmental mechanisms, and considerable groundwork was necessary to reach this point (Inhelder, 1989, ix–x). Part of the groundwork was done by the stages of cognitive development. In fact, Piaget regarded the stages as an essential analytical tool for investigating developmental mechanisms:

Why does everyone speak of stages? One tries to construct stages because this is an indispensable *instrument for the analysis of formative processes.* Genetic epistemology attempts to envisage the construction of mental functions, and stages are a necessary instrument for the analysis of these formative processes. But I must vigorously insist on the fact that stages do not constitute an aim in their own right. I would compare them to zoological or botanical classification in biology, which is an instrument that must precede analysis (Piaget, 1977, 817 *Author’s*).

For Piaget, then, the stages were a means to an end, not an end in themselves. However, a heuristic can give a false impression. A classificatory tool facilitating the analysis of developmental mechanisms might be regarded as a convenient convention used to parse cognitive development into artificial divisions. Indeed, the variety of stage models advocated by Piaget lends support to this impression. For Piaget, however, the stages are not simply convenient conventions; they describe natural divisions in cognitive development (Piaget, 1977, 814–5). Moreover, Piaget’s analogy with classificatory systems in biology makes it clear that he saw no inherent contradiction in the notions of an analytic tool, on the one hand, and natural divisions, on the other.

Finally, the stage models characterize cognitive development in terms of cognitive structures that undergo an invariant sequence of transformations. The degree of abstraction in the stage models is thus extremely high, and this reflects Piaget’s interests not in the psychological subject but in an epistemic subject. For Piaget (1970, Sect. 13 & pp. 138–140), the epistemic subject has structural and functional aspects: structurally, it is synonymous with the cognitive core common to all subjects at a particular stage of cognitive development, the cognitive structures that characterize a particular stage; functionally, it is the motor imminent in cognitive structures driving the construction of new structures.

## *Décalage*

Experimental investigation of cognitive development revealed systematic asynchronies in the performance of certain tasks. The conservation tasks are a classic example. Theoretically, tasks involving the conservation of matter, weight and volume can be mastered using the same cognitive structure; consequently, there is no structural reason why children should not grasp them synchronically. Yet it has been shown that mastery of the conservation of volume systematically lags behind mastery of the conservation of weight, and mastery of the conservation of weight systematically lags behind mastery of the conservation of matter. For systematic delays in cognitive development such as those found in the conservation tasks, Piaget coined the term ‘*décalage*’ and distinguished thereby horizontal from vertical *décalage*. Horizontal *décalage* designates the systematic delays in performance of tasks that are isomorphic from a structural point of view. Vertical *décalage*, on the other hand, designates systematic delays in the reconstruction of structures already present at lower stages in terms of the operations emerging at higher stages. For example, when the group of displacements, which is already acquired for actions at the sensorimotor stage, is reconstructed in terms of representations at higher stages of development. In short, horizontal *décalage* designates an intra-stage delay whereas vertical *décalage* designates an inter-stage delay (Piaget, 1977, 816; Kesselring 2009, 374).

Vertical *décalage* is easily explained without going beyond the framework of Piaget’s stage models. The stages are sequential and each new stage provides a new medium in terms of which structures already existing at the previous stage are reconstructed. Horizontal *décalage*, in contrast, cannot be explained in terms of the reconstruction of a pre-existing structure in a new medium after transition to a higher stage since it occurs within a stage, nor can it be explained in terms of differences in structures within a stage since it designates systematic delays in the performance of structurally isomorphic tasks. From the point of view of Piaget’s stage theory, horizontal *décalage* is thus an anomaly.

### Horizontal ***Décalage*** Explained

In 1968, Piaget invoked the ‘resistance of the objects’ to structuration in order to explain horizontal *décalage* (Montangero, 2000, 509). In doing so, he redressed a bias inherent in the stage models. Hitherto, cognitive development is described in terms of the sequential transformations of characteristic cognitive structures; the point of view of the epistemic subject thus dominates the stage theory. However, there are two poles in cognition—the knower and the known. The resistance of objects thus reintroduces the effects of the hitherto neglected pole into the models.^1^ Nevertheless, as an explanation of the observed systematic delays, it is still unsatisfactory since it does not explain how objects resist assimilation to cognitive structures. Attempts have been made to address this shortcoming.

One attempt draws on a distinction Piaget made between form and content. We have met this distinction already in the context of vertical *décalage*: a structure—the group of displacements, for instance —is a form that is reconstructed in terms of new contents—spatial representation rather than sensorimotor activity—as cognition transitions to higher stages of development. However, form and content are relative rather absolute concepts, according to Piaget, so that forms structure contents but can also constitute content for other forms. A test person presented with a task in an experimental design is thus required to assimilate a given content to a cognitive structure. Prior to the successful deployment, however, the content, which is already replete with forms, has to be differentiated and organised appropriately. On the one hand, cultural, socioeconomic and individual factors can facilitate or hinder successful assimilation through preliminary organisation of a content. On the other hand, some content is inherently more complex than others and thus requires more assimilatory effort before a structure can be successfully deployed; for instance, the degree of complexity inherent in the conservation experiments is thought to increase progressively through matter, weight and volume. According to this view, the resistance of the objects thus has a twofold source: the inherent complexity of the content itself and the preliminary organisations imposed by cultural, socioeconomic and individual factors (Montangero, 2000, 516–7).

### ***Décalage*** and Cognitive Growth

Despite deficits in explaining it, the phenomenon horizontal *décalage*, once an anomaly, proved not only to be beneficial in reinstating the effects of the object, the hitherto neglected pole of cognition, in the stage models but also in explaining cognitive growth. Equilibration is one of the mechanisms of cognitive development that formed the focus of Piaget’s later work, and, in general terms, it has already been mentioned that Piaget regarded cognitive structures as equilibria. Horizontal *décalage* is not a mechanism of cognitive development; nevertheless, it plays a vital role in the construction of cognitive structures in conjunction with equilibration. According to genetic epistemology, a subject interacts with its environment in cognitive activity, thereby conferring meaning on parts of it by virtue of the interaction. In particular, parts of a subject’s environment are incorporated into behavioural or conceptual schemes, the form governing an interaction, and these schemes, in turn, adapt to the particulars of the part of the environment being incorporated. Piaget denoted these processes ‘assimilation’ and ‘accommodation’, respectively. The cognizing subject and its environment, therefore, forms an open system, in which the circular interaction—scheme, assimilation, and accommodation—is held in a dynamic state of equilibrium by equilibration (Piaget and Garcia 1991, 128ff). The environment should, however, be more broadly construed than just external objects and events since schemes can also assimilate other schemes. In fact, schemes assimilate and accommodate each other reciprocally, and Piaget believed that *décalage* played a vital role in cognitive growth during this reciprocal assimilation.

Through various cognitive activities, schemes often originate in different fields of knowledge and proceed to develop in comparative independence of each other before eventually being synthesised to form operations and larger systems, such as cognitive structures. However, the independent lines of development and the different developmental speeds of the schemes give rise to disequilibria during reciprocal assimilation, which are eventually overcome and replaced by new, qualitatively different, states of equilibrium through the action of equilibration (Piaget, 1985, 3). In other words, *décalage* stimulates cognitive development by producing the disequilibria that precipitate re-equilibration and the formation of new, more stable cognitive structures (Montangero, 2000, 509). In conjunction with equilibration, then, horizontal *décalage* was no longer an anomalous phenomenon but became an integral part of the theory of cognitive growth (Piaget, 1985, 7).

## Criticism of Piaget’s Stage Theory

Having briefly reviewed the development of Piaget’s stage theory, characterised the stages of cognitive growth, and the role they play in genetic epistemology, it is time to consider issues surrounding it. Apart from the coherence of the theory, which has already been discussed in the previous section, the term ‘stage’ has attracted criticism. It implies a certain discontinuity in development, in which things at one stage are more alike than things in neighbouring stages; for instance, caterpillar-cocoon-butterfly are stages in the life-cycle of a butterfly. However, the characteristics of stages set out above introduce continuity into cognitive development, and this is grist to the mill for those who criticise the stage models (Kesselring, 2009, 4). Continuity of structures goes hand-in-hand with integration, for example, and the phases preparation and culmination comprising consolidation as well as the transition from disequilibrium to equilibrium through equilibration already evoke continuity rather than abrupt changes. Nevertheless, it can be argued that the contradiction between discontinuity and continuity for the stage models is more apparent than real. By distinguishing between functional and structural aspects in cognitive growth, continuity can be attributed to the functional aspects while discontinuity is reserved for structural aspects. Thus, the transition from disequilibrium to equilibrium by means of equilibration is continuous from a functional point of view, but, from a structural point of view, the new equilibrium corresponds to a new structure, which is qualitatively different from its predecessors and allows manifestly different cognitive behaviour (Piaget and Garcia 1991, 138–9; Inhelder 2000, 28).

Whereas continuity and discontinuity can be reconciled through a theoretical distinction between structural and functional aspects of cognitive growth, *décalage* makes a similar reconciliation of observable behaviour and stages difficult. From the theoretical point of view, each stage is clearly characterised by cognitive structures. However, *décalage* stimulates cognitive growth via disequilibria: cognitive schemes develop in comparative independence and at different speeds in disparate fields of knowledge (see Piaget and Garcia 1991, 140); since observable cognitive behaviours follow these independent lines of development, they will not typically be representative of a single stage of cognitive development. From the empirical point of view, then, cognitive behaviours indicative of the cognitive structures characteristic of a particular stage of development will emerge at different ages and progress at different rates. The stages so clearly delimited in theory through structural discontinuity, therefore, lose their clear-cut boundaries in empirical research since *décalage* introduces variation in observable cognitive behaviours.

Critique has also been levelled at the high degree of abstraction mentioned at the end of Sect. 1.1.1 above. Science seeks generality, and abstraction goes hand-in-hand with generality; at first sight, the high degree of abstraction characteristic of Piaget’s stage models would therefore appear to attest to their scientific credentials. However, each child is born with a particular biological disposition, is raised by particular parents or guardians in a particular socioeconomic milieu in a particular culture at a particular time and is tested under certain conditions. Moreover, empirical evidence indicates that these factors influence cognitive development and experimental results.

Cross-cultural studies have shown cultural differences in the performance of Piagetian cognitive tasks. Dasen (1975), for instance, contrasted the performance of children from Eskimo [sic], Australian Aboriginal and Ebrié of the Ivory Coast communities in Piagetian cognitive tasks designed to assess the attainment of the concrete-operational stage. Using Berry’s ecocultural scale for subsistence groups, he classified the Eskimo and Australian Aborigines at the low food-accumulating, migratory, hunting and gathering, low population density end of the scale and the Ebrié Africans at the high food-accumulating, sedentary, agricultural, high population density end. He discovered that Eskimo and Aboriginal children acquired spatial concepts more rapidly than their Ebrié counterparts whereas the latter acquired conservation concepts more rapidly than the former, though not unequivocally. As hunter-gathers, spatial skills are vital to the subsistence of Eskimos and Australian Aborigines, whereas production, accumulation and exchange of food are of vital importance for the Ebrié as sedentary farmers. Besides showing cultural differences in the rate of cognitive development, Dasen’s results, thus, partially confirmed the dependency of cognitive development on the practices proper to the culture in which children are socialised, as well (see also Dasen 1984). Comparisons of children raised in non-Western cultural groups with their Western counterparts have also shown that cognitive development is less delayed in those raised in urban rather than rural environments. And some studies even show that children raised in rural environments only manage to perform a portion of the tasks characteristic of the concrete-operational stage. Nevertheless, the time lags have been shown to decrease with increasing contact with Western culture and technology and vanish completely when children from non-Western cultural groups are raised in a Western context (Dasen and Heron 2000, 81). Research also shows that socioeconomic factors have a lasting effect on cognitive development. Lloyd (1971), for example, found that the cognitive advantages of economically and intellectually privileged socialisation, which manifested early in the cognitive development of Yoruba Ss children, did not diminish in the course of cognitive development. Though the results are not systematic, differences in cognitive development are also attributable to schooling (Dasen and Heron 2000, 81–2). Furthermore, the physical and social setting in which a child lives, customs of childcare and rearing, and the psychology of childcare, or ethnotheories of development, comprise a developmental niche affecting the cognitive development of children (Dasen and de Ribaupierre 1987, 799–800). In addition, a large amount of inter-individual variability in children between 6 and 12 years of age has been discovered which was thought to be due to vicarious processes within the individual that develop differently in different subjects and that are differentially elicited in different situations (Dasen and de Ribaupierre 1987, 804).

In summary, empirical research indicated that cognitive development, like many human traits, is influenced by a complex interplay of biological, individual, socioeconomic and cultural factors; however, the degree of abstraction inherent in the stage models was a cause of concern amongst developmental psychologists since it does not account for the observable differences in cognitive development due to these factors (Kesselring, 2009, 374). The stages are characterised by cognitive structures, but empirically children master the tasks designed to assess the acquisition of the cognitive structures characteristic of any one stage at different rates. Moreover, it has also been shown that training in the performance of a class of tasks related by a specific cognitive structure has a positive impact on the performance of the tasks in question without affecting the performance of structurally related tasks (Kesselring, 2009, 374). Although these findings can be integrated into the theory of genetic epistemology via *décalage*, the stage theory itself does not explain them.

Methodological artefacts are another source of variation in experimental results. In contrast to the standardised procedures preferred by most psychologists, critical exploration lies at the heart of the clinical method developed for Piagetian research into cognitive development. The experiment is based on a task designed to reveal a particular cognitive structure; however, the task itself only forms the point of departure for an extensive, in-depth dialogue between the experimenter and the test child, in which the experimenter’s questions are prompted by the actions and responses of the child. Through such a dialogue, a skilled interviewer can probe the leads provided by the child’s reactions in order to investigate the cognitive structures underlying them. Adaptability to many settings is one of the advantages of the clinical method; however, its major drawback is the dependency of the results on the quality of the communication between experimenter and subject as well as the competence of the experimenter. On the one hand, language and cultural difference can diminish the quality of communication; on the other hand, the experimenter has to be fully conversant with Piaget’s theory in order to guide real-time hypothesis-testing during the experiment (Dasen and Heron 2000, 88). In contrast to the difficulties set out in the next paragraph, the effects of these methodological artefacts on experimental results can be mitigated through adequate training of indigenous experimenters.

We are all familiar with those retrospective reflections on our actions which begin ‘if only I had …’. They are often an expression of regret that a capacity to act in a certain way was not in fact used. In the heat of the moment, for example, it doesn’t occur to tourists to take pictures of pickpockets openly plying their trade although, or perhaps because, they have been taking holiday snaps during a sight-seeing tour of the city. The example illustrates the gulf between competence and performance; no-one would contest the camera-wielding tourists’ ability to take pictures of the pickpockets, yet for some reason it does not occur to them to do so. In the experimental situation, it is also pertinent to distinguish between competence and performance. Accordingly, competence corresponds to the ideal realm of cognitive structures whereas performance corresponds to the behaviour and responses to questions actually demonstrated in the experimental situation. The example also illustrates how the relationship between the two is complex. Many factors prevailing at the time of the experiment can intervene in the translation of a competence into a performance; performance is therefore an unreliable indicator of competence. Nevertheless, the experimenter only has performance to go on and must rely on his/her skill as an interviewer in order to elicit competence from performance (O’Brien and Overton 1982; Overton and Newman 1982; Dasen and Heron 2000, 90ff).

In summary, Piaget’s stage theory is extremely abstract. It models the cognitive development of an epistemic subject—the common core and developmental motor, that is, of cognitive structures shared by all subjects at a particular stage in development—in terms of an invariant sequence of cognitive structures. With this degree of abstraction, the stages are clearly delimited, and the discontinuity of transitions from one stage to the next are clearly marked by the changes in cognitive structures. In contrast to the rarefied atmosphere of theory, however, catalytic^2^ transformations from one stage to the next are clouded in an empirical fog. Systematic asynchronies in structurally isomorphic tasks were discovered in observable cognitive behaviour. Piaget acknowledged this type of variability and integrated it into the framework of genetic epistemology as horizontal *décalage*: on the one hand, he attributed horizontal *décalage* to the resistance of objects; on the other hand, he regarded it as a vital stimulus for cognitive growth. Moreover, experimental subjects, unlike epistemic subjects, are embedded in biological, psychological, socioeconomic, and cultural contexts, which along with situative factors impact on the performance of cognitive tasks during experimentation. Thus, the observable cognitive behaviour of real subjects can still diverge considerably from the behaviour expected of an epistemic subject at a particular stage of cognitive development even after taking into account delays due to the resistance of objects.

One of the enduring criticisms of Piaget’s stage theory is that it is not able to account for the observable differences in cognitive development because it is too general and monolithic (Kesselring, 2009, 374). *Décalage* was Piaget’s concession to observable asynchronies in cognitive development. However, the resistance of objects only accounts for a portion of them, and as experimental results demonstrating variability in cognitive development began accumulating in the 1970s, researchers started abandoning Piaget’s stage models in what became known as the ‘Crisis of Variability’ (Rose and Fischer 2009). However, should the theory’s scientific credential be sacrificed for a less general, less monolithic, domain-specific or modular theory of cognitive development more in line with the variation observable in empirical research? Or, is the disparity between the stage theory and empirical variability typical of the relationship between theory and experiment in the sciences? The next section will investigate the relationship between Piaget’s stage theory and empirical research from the perspective of fuzzy structuralism.

### Fuzzy Space

Whilst conceding that the overall picture is more complex than originally thought, Dasen believed that his summary of Piagetian research from 1972 still largely reflected the state of research in 1981:

- The qualitative aspects of the theory (the sequence of stages and substages, their structural properties, the types of explanations given by respondents) have found support in a great majority of studies;
- The horizontal décalage (e.g., the sequential appearance of conservation, weight, and volume) characterizes sample means, but are not found in all individual subjects;
- The quantitative aspects (the rate of progress through the stages, or the chronological age at which these are attained) show considerable variations. (Dasen and Heron 2000, 72)

In his summary, Dasen distinguishes between qualitative and quantitative aspects of Piaget’s theory. On the one hand, we have seen how the stages are characterised by cognitive structures, and the clinical method developed by Piagetian researchers is designed to elicit them. On the other hand, cognitive structures develop over time. The former emphasises the qualitative aspects, focusing on the sequential transformation of the structures characterising the stages; the latter, in contrast, is quantitative, assigning age ranges to the sequence of stages. Dasen sees the qualitative aspects of the theory supported by research; however, the variation in the quantitative results give grounds for concern since it may not be possible practically to determine the developmental stage of children unequivocally. Inuit children, for instance, may well be at the concrete-operational stage of cognitive development according to spatial but not conservation criteria, and vice versa for their African counterparts. Contradictory messages therefore seem to emanate from the qualitative and quantitative aspects of Piaget’s stage theory, and in order to get to the roots of the Crisis of Variability it is necessary to unearth the connection between theory and empirical research.

Whereas Piaget’s stage theory has already been set out in the previous section, the empirical methods used in its verification have not. To remedy this deficit, the methods Dasen used in his cross-cultural studies will be outlined.

**Fig. 1:**
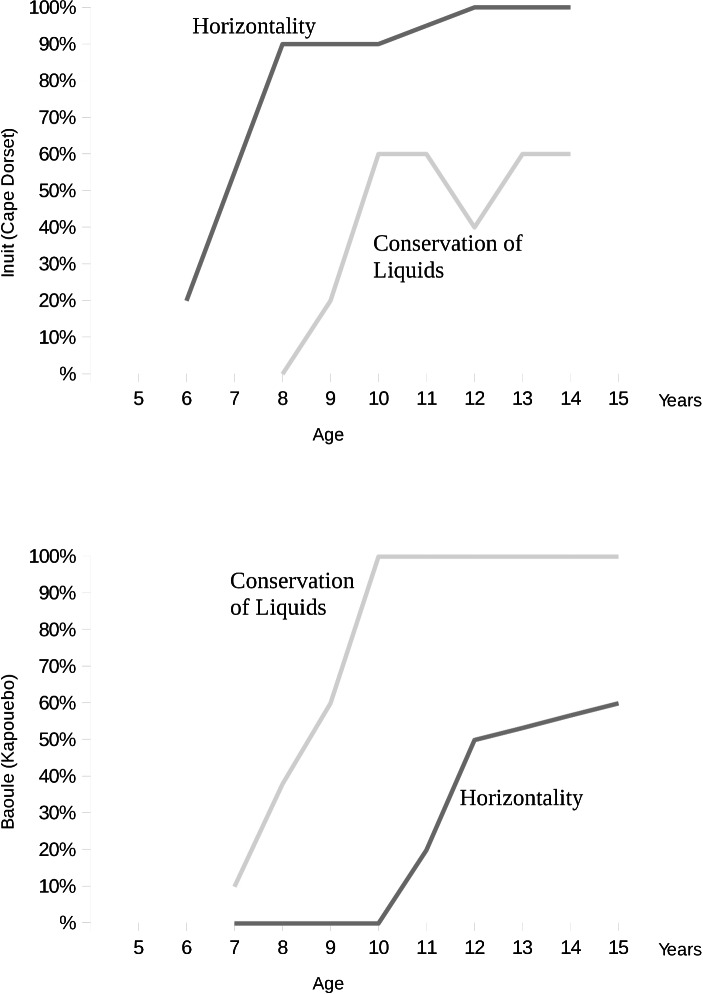
Experimental Development Curves. The developmental curves show the percentage of children in age groups from two different ethnic groups performing at the concrete-operational stage in Piagetian tasks on horizontality (assessment of the surface level of liquids in containers resting at different angles) and conservation of liquids. (After Dasen 1984, 410)

First, a particular cultural group is chosen and classified according to Berry’s ecocultural scale; a sample of children covering an appropriate age range for the stage under investigation is then selected and characterised according to age, gender, schooling etc. Age groups are then constructed, and the number of children in each group is determined. Standardised tasks designed to elicit the cognitive structures characteristic of the cognitive stage under investigation are then implemented using the clinical method, and the results are recorded in a frequency table divided into three stages according to success (failure, partial success and complete success). The results are then presented in developmental curves for each of the tasks investigated. A developmental curve plots the age groups (x-axis in Fig. 1) against the proportion of children in a particular age group successfully completing the task (y-axis in Fig. 1).

**Fig. 2:**
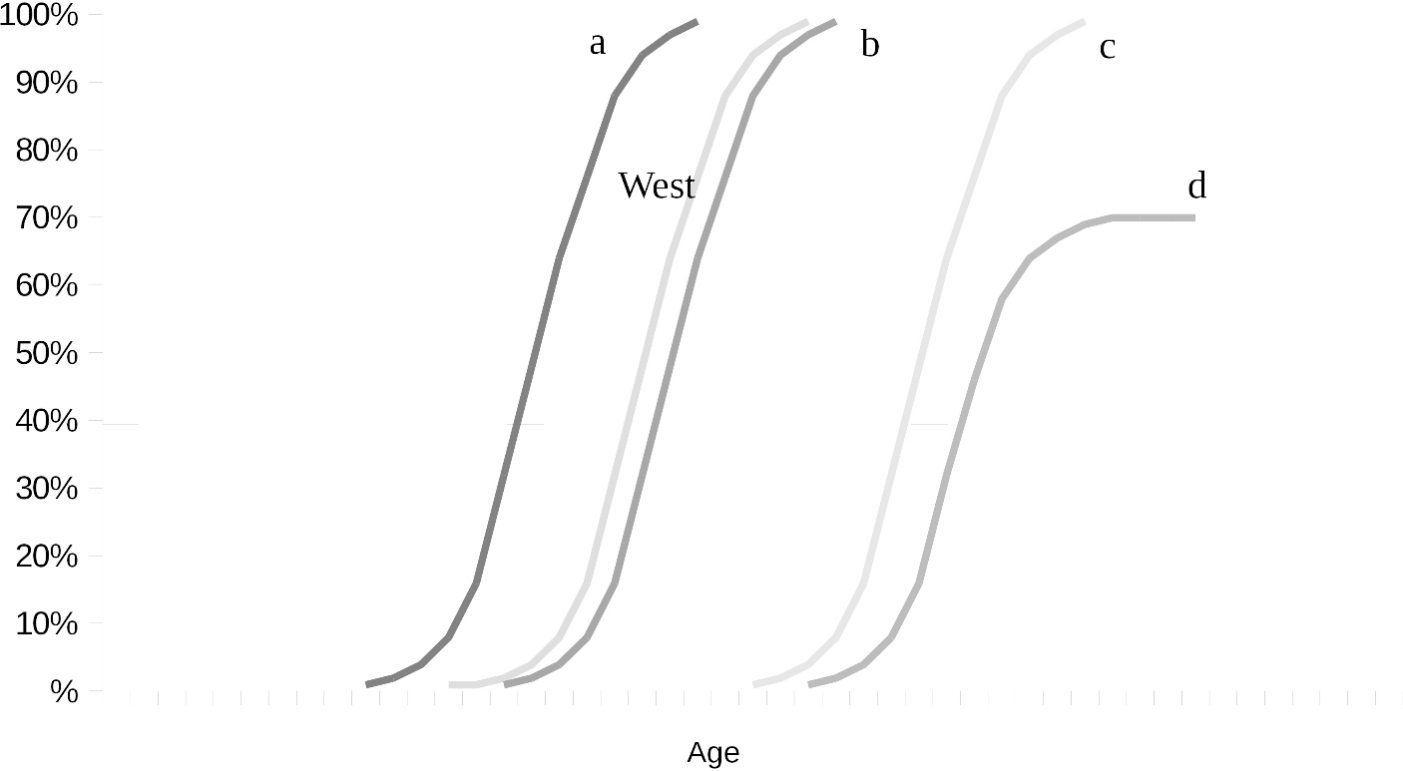
Theoretical Development Curves. The curves represent the percentages of participants at a given age performing a particular task at the required stage. The curve denoted ‘West’ depicts the performance of children with a Western, technological background; curves to the left (a) or right (b), (c), (d) represent possibilities for developmental curves derived from cross-cultural studies (After Dasen and Heron 2000, 80)

Within a particular cultural group, there is usually considerable variability in the ages at which behaviour characteristic of a cognitive structure manifests. Although the majority exhibit the characteristic performance at approximately the same age, there are always precocious children and late developers. Nevertheless, the proportion of children in any age group demonstrating the performance characteristic of the stage increases with age, thus giving rise to curves over a developmental age range like those in the Fig. 1. Intercultural variation in cognitive development is then investigated by comparing the developmental curves of different cultural groups for a particular task. Generalised results of cross-cultural comparisons can then be depicted in theoretical development curves (Fig. 2). Research has shown that the cross-cultural differences are not qualitative but quantitative; the theoretical development curves thus depict a generalised typology of quantitative variation occurring in cross-cultural comparisons by means of similarly shaped curves. The generalised shape depicts the typical increase in the proportion of children able to perform a cognitive task with increasing age. The form of the curves is thus representative of the development of cognitive structures over time, while displacements along the x-axis represent the quantitative variations in the age at which these structures develop.

At a theoretical level, cognitive development is represented by the sequential transition from one cognitive structure to another as the epistemic subject progresses from one stage to the next. At an empirical level, the performance of individual children at specific cognitive tasks is investigated. However, even the empirical level is not completely devoid of theory. On the one hand, the cognitive tasks are designed to reveal particular cognitive structures through a child’s cognitive performance; they are thus inspired by Piagetian theory. On the other hand, the cross-cultural comparisons of the cognitive performance of children are made possible by the classification of cultural groups according to Berry’s ecocultural scale. Moreover, the cognitive development of the epistemic subject is qualitative, and the sequential transitions of cognitive structures translates into age for real subjects; however, the studies are not longitudinal. The developmental curves show the proportions of children in a particular age group of a sample group able to perform a particular cognitive task. Individual cognitive development over time is therefore replaced by the proportional performance of children in age cross-sections of the sample group. Since cognitive development is sequential and the sequence manifests in time, a larger proportion of success at a task can be expected in a group of older children than in a group of younger cohorts. Theoretical considerations therefore legitimise the substitution of age cross-sections through a sample group for the actual cognitive development of children over time.

Theory clearly permeates the experimental design and provides the framework in which the results are presented and interpreted. However, permeation is not a one-way street. It has already been mentioned how meticulous observation of his own children led Piaget to amend his early stage theory of cognitive development, and horizontal *décalage* was initially an empirical anomaly before becoming an integral part of the theory as a developmental stimulus. Piaget thus developed and modified his theory further to accommodate observations and empirical findings.

**Fig. 3:**
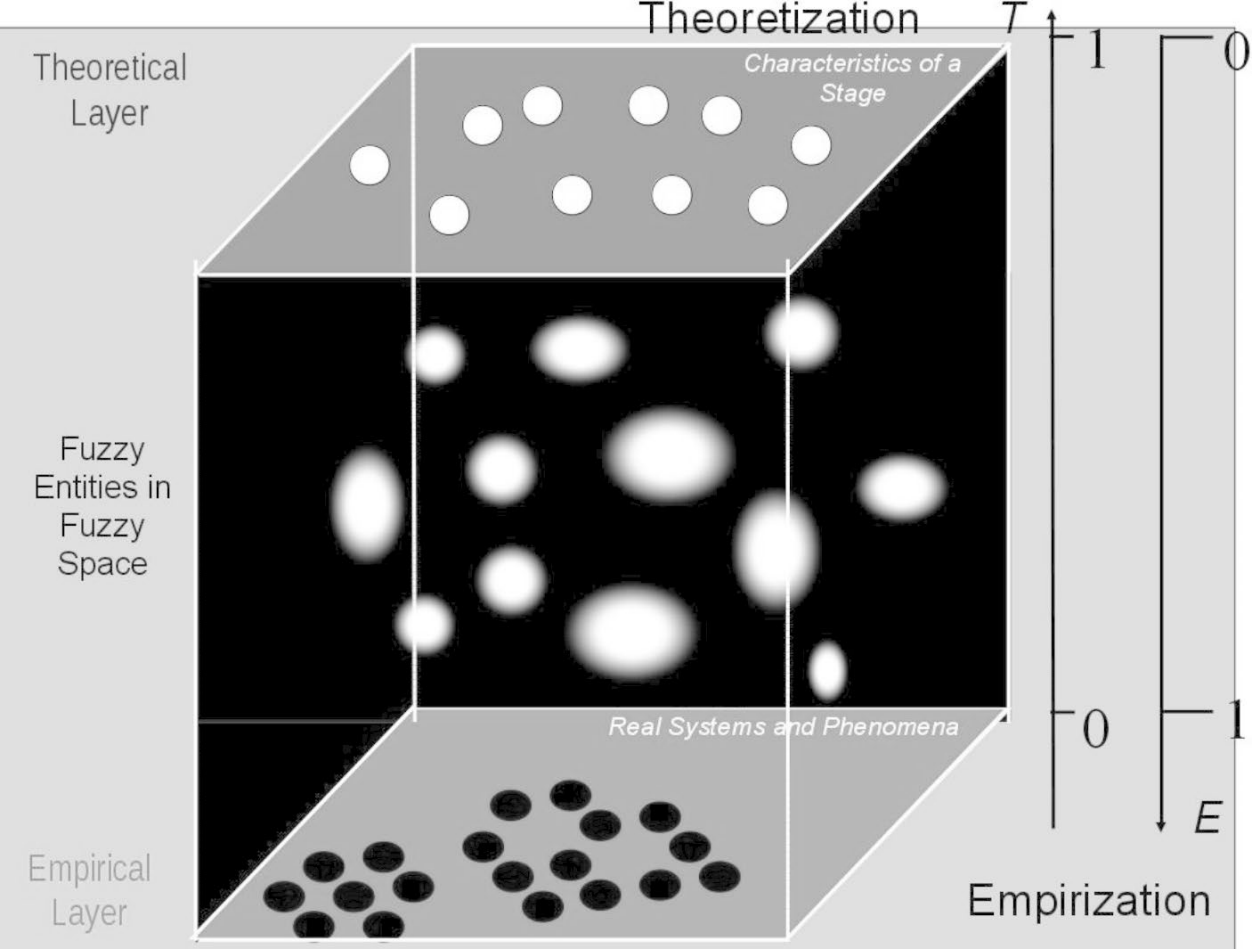
Fuzzy Space between Theoretical and Empirical Layers. T(c) (Theoretization) and E(c) (Empirization) are functions of concepts (or perceptions) c. They map the opposing theoretical and empirical perspectives on c to values ranging from 0 to 1. Accordingly, E(c)=1 and T(c)=0 are completely empirical and T(c)=1 and E(c)=0 are completely theoretical. (After Seising 2009, Fig. 1.16 & 1.17)

Rudolf Seising (2007; 2009; 2013) has proposed a model for scientific theories and experimental findings in terms of fuzzy space (see Fig. 3), which provides a useful framework in which to interpret the permeation of theory and empirical findings set out above. The theoretical layer is populated by an epistemic subject that develops cognitively in an invariant sequence of stages characterised unequivocally in terms of cognitive structures. In the theoretical layer, the stage theory is abstract and qualitative; however, in the process of empirization it becomes increasingly concrete and quantitative. On the one hand, the epistemic subject gives way to cultural, socioeconomic, psychological, and biological individuals performing particular cognitive tasks under specific conditions as it is progressively embedded in reality. Moreover, the various layers of embedding and the inherent difficulty of the tasks have been shown to influence the cognitive performance of individuals, and their effects are reflected in the variability of quantitative results. The empirization of the stage theory thus leads to quantitative variation in the rate of progression through the stages measured in the chronological age at which cognitive abilities manifest. On the other hand, quantitative variation diminishes progressively in the process of theoretization. Biological, psychological, socioeconomic, cultural etc. influences along with the resistance of the objects all contribute to the quantitative variation in the frequency tables. By comparing the development curves for specific tasks, however, it is possible to abstract from the quantitative variation due to other factors in order to focus on the variation due to the factor under investigation. By abstracting from the specific influences of particular factors, theoretical developmental curves can be generated, and the horizontal translations of these curves represent a general typology of quantitative variation; but they also show development through the increasing proportion of children able to perform tasks at the required level as a function of age. Finally, age-related development is replaced by qualitative sequential transformations of cognitive structures in an epistemic subject while quantitative variation becomes a developmental stimulus in the form of *décalage.*

### Stages in Fuzzy Space

The term ‘stage’ is used in several different ways in conjunction with Piaget’s theory. An empirical stage is a classification of performance at a particular task according to success; a theoretical stage, on the other hand, is characterised by specific cognitive structures. In ordinary usage, however, ‘stage’ denotes the level of cognitive development achieved by individuals or groups rather than the epistemic subject but without necessarily referring to any specific tasks;^3^ ‘stage’ in ordinary usage therefore does not conform with usage in the theoretical or empirical layers.

It has already been mentioned that each stage is characterised by certain structures, and the epistemic subject is the cognitive core of all individuals at a particular level of cognitive development. In the theoretical layer, then, an epistemic subject is always unequivocally at a particular stage. However, it has already been mentioned that the catalytic transformations in cognitive behaviour corresponding to the transitions from one theoretical stage to the next are obscured in an empirical fog as soon as the rarefied atmosphere of theory is left behind. The metaphor alludes to the quantitative variation in the chronological ages for the attainment of and rates of progression through the stages due to the empirization of the stage theory. However, good metaphors have suggestive powers that fuel the imagination. The indistinct details and blurred contours the imagery of fog conjures up are reminiscent of fuzzy sets, and I will explore the use of the term ‘stage’ between theory and experiment in the light of fuzzy sets in the next subsection.

#### Fuzzy Set Theory

Fuzzy sets were introduced into the field of science in 1965 by Lotfi A. Zadeh, who was professor at Berkeley in the Department of Electrical Engineering and Computer Science at the time. He was already a pioneer in systems theory when he recognised a gap that ‘reflects the fundamental inadequacy of the conventional mathematics—the mathematics of precisely-defined points, functions, sets, probability measures, etc.—for coping with the analysis of biological systems … which are generally orders of magnitude more complex than man-made systems’ (Zadeh, 1962, 857). His search for possible ways of bridging the gap between abstract mathematical theories and such systems led him to consider a ‘radically different kind of mathematics, the mathematics of fuzzy or cloudy quantities which are not describable in terms of probability distributions’ (Zadeh, 1962, 857).

Two years later he established the theory of fuzzy sets and systems (Zadeh, 1965a, b) as a mathematical theory of ‘membership functions’ for classes with unsharp boundaries (Seising, 2007): fuzzy sets ‘are not classes or sets in the usual sense of these terms, since they do not dichotomize all objects into those that belong to the class and those that do not.’ He introduced ‘the concept of a fuzzy set, that is a class in which there may be a continuous infinity of grades of membership, with the grade of membership of an object *x* in a fuzzy set A represented by a number *µ*_*A*_(*x*) in the interval [0,1].’ (Zadeh, 1965a; Seising, 2007).

**Fig. 4:**
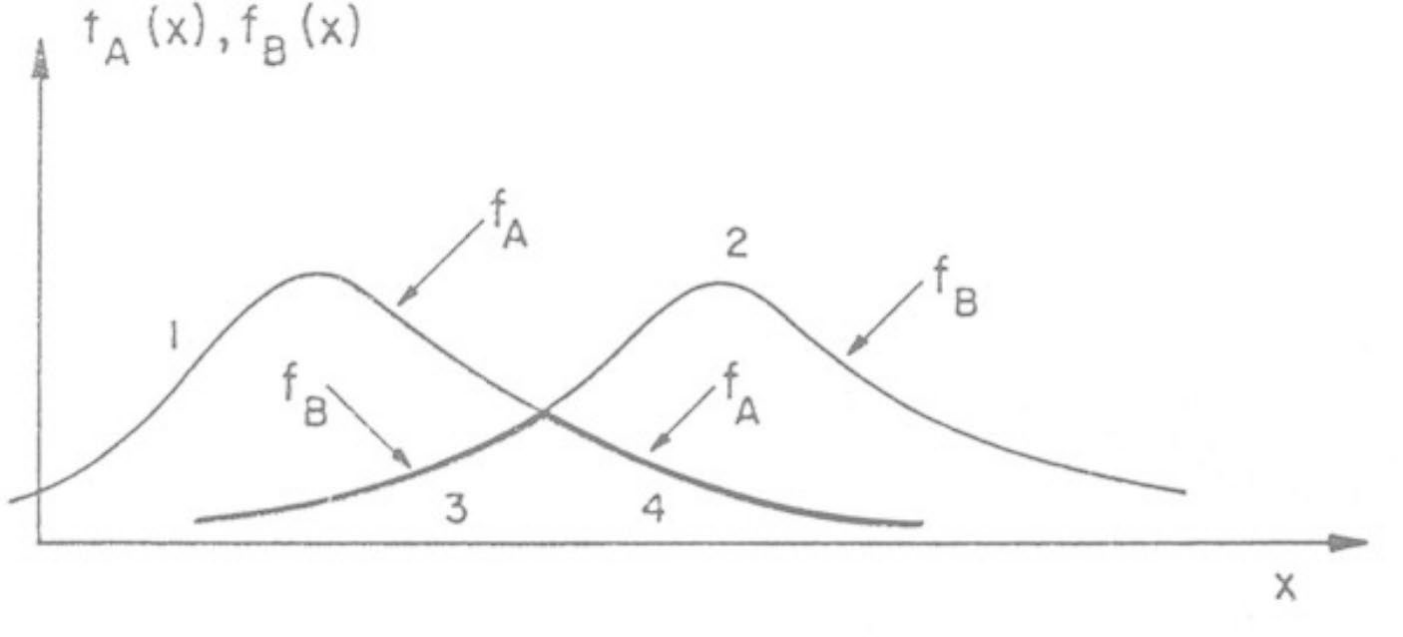
Intersection and Union of Fuzzy Sets. The graph shows the intersection f_A∩B_ (x) and union f_AUB_ (x) of fuzzy sets A and B (Zadeh 1965a, 342). In this article Zadeh used f to refer to the membership function. Later he used the Greek letter µ

For the mathematical theory of fuzzy sets, he also generalized the concepts of operations on classical sets. He defined equality, containment, complementation, intersection and union (see Fig. 4) relating to fuzzy sets A, B in any universe of discourse X as follows (for all *x* ∈ X):

- A = B if and only if *µ*_*A*_(*x*) = *µ*_*B*_(*x*),
- A⊆B if and only if *µ*_*A*_(*x*) ≤ *µ*_*B*_(*x*),
- ←A is the complement of *A*, if and only if *µ*_←*A*_(*x*) = 1- *µ*_*A*_(*x*),
- A⋃B if and only if *µ*_*A*⋃*B*_(*x*) = max (*µ*_*A*_(*x*), *µ*_*B*_(*x*)),
- A⋂B if and only if *µ*_*A∩B*_(*x*) = min (*µ*_*A*_(*x*), *µ*_*B*_(*x*)).

In the early 1970s, Zadeh wanted to apply his theory of fuzzy sets in non-technical fields like linguistics and psychology. He initiated interdisciplinary discussion with the psychologist Eleanor Rosch and the linguist George Lakoff, who were also working at Berkeley at the time. Rosch’s research, especially, showed that concept categories are graded; consequently, she argued that concepts are not adequately represented by classical sets.

Clearly, Rosch’s research also has implications for psychological concepts, especially those in everyday use. If stages of cognitive development, for example, are graded concepts, then they cannot be adequately represented by classical sets. Later, Rosch (1973) went on to develop a ‘prototype theory’ on the basis of empirical studies. The theory maintains that people categorise objects in the real world by comparing them with prototypes, and word meanings are formed from prototypical details and scenes, before being incorporated into lexical contexts depending on the context or situation. In the present context, the way stages of cognitive development are used in ordinary language indicates that certain types of cognitive behaviour have become prototypical of behaviour at particular stages of cognitive development.

#### Stage, a Fuzzy Concept in Fuzzy Space

In ordinary usage, the term ‘stage’ is applied to individual children or groups of children in the process of cognitive development; however, it is not being used in either the theoretical or empirical sense since neither the cognitive structures characteristic of a stage nor the successful performance of a particular cognitive task are serving as criteria for its attribution. This concept of stage thus hovers somewhere in the fuzzy space between theory and experiment in Seising’s model. Moreover, certain kinds of cognitive behaviour are prototypical when attributing a stage of cognitive development in ordinary usage. Nevertheless, performance at particular cognitive tasks, regardless of whether they are considered prototypical or not, can only provide empirical evidence that a particular stage of cognitive development has actually been achieved. Moreover, no matter how overwhelming the empirical evidence appears to be, it will never be completely conclusive. ‘Stage’ in ordinary usage thus appears to be a graded concept. In my opinion, these considerations warrant attempting to operationalise such stages in fuzzy space in terms of fuzzy sets. In order to simplify matters, however, I will confine myself to the attribution of such stages to individual children.

A universe of discourse can be defined as follows:

C={Children in the process of cognitive development}

Empirical evidence is required to attribute a stage of cognitive development to a child; however, assuming a child performs a task t_i_ at the concrete-operational level, the child has fulfilled a criterion for the particular stage but cannot be judged to have attained it on the basis of this success alone. Theoretically, each stage is characterised by a number of cognitive structures, and the tasks are designed to elicit empirical evidence for their attainment. However, task performance is subject to quantitative variation, since *décalage* coupled with cultural, socioeconomic, situative, psychological, biological, etc. factors influence performance. Hence an Inuit child, for example, may well be at the concrete-operational stage as far as spatial tasks are concerned but not yet for conservation tasks and vice versa for African children of the Baoulé.

Although successful performance at a single task alone is not sufficient grounds for attributing a stage, it might be argued that several tasks fare better. However, preferences in cognitive structures have no theoretical basis. Regarding some structures as prototypical would therefore be artificial from the theoretical point of view, and it would run the risk of introducing bias and hegemony into the attribution of stages (see Dasen 1984). Equally, treating unsuccessful performance at a particular task as a counterexample unduly weights failure. To avoid bias, hegemony and weighting failure too heavily, performance at tasks related to all cognitive structures characteristic of a stage must be taken into account. Fortunately, the developmental curves suggest a way forward.

Developmental curves plot the proportion of children in a particular sample group successfully performing the task in question against age. Although each child of the sample group only features in his/her age group in the developmental curve according to his/her success or failure at the task in question, the relationship between the proportion of successful performances and age for the sample group as a whole could be used as a measure of the degree of success any child in a sample group has at performing the task. For any child, the degree of success would then be a function of age; moreover, it would provide a measure for the membership of any child in the class of successful task performers, which could be expressed formally as follows:

Child c is a member of the class of task performers T_i_ µ_Ti_ (c), where µ_Ti_ ∈ [0,1]^4^

A stage is characterised by a number of cognitive structures; theoretically, success in all of the tasks attesting to these structures is therefore required in order to warrant attributing a stage of cognitive development to a child. However, this is an ideal that cannot be realised in practice. Moreover, success at performing tasks varies empirically; a higher proportion of children within a particular age group may be more successful at, say, task t_i_ than t_j_ and more successful at t_j_ than t_k_, for instance. A particular child in the sample age group may therefore be able to perform t_i_ and t_j_ but not t_k_; indeed, a child may be able to perform all of the tasks but one successfully, and to refuse to acknowledge this achievement by the attribution of a stage would seem unduly harsh. Via degrees of success, on the other hand, performance at all the tasks would be taken into account while avoiding the excessive weight given to a single failure at any particular task. Rather than assessing performances dichotomously with success and failure, a higher degree of success at t_i_ than at t_j_ and at t_j_ than t_k_ could be attributed to a child at a particular age; a stage could then be assigned on the basis of degrees of success rather than actual successes and failures.

In general, a number of cognitive structures characterise a stage, which are investigated empirically by a number of experimental tasks, t_i_ for i = 1…n. Let T_i_ denote the class of task performers for a particular age sample group, and S, the stage performers. Ideally, the class of stage performers is constituted by those children who are able to perform all of the tasks t_i_ for i = 1 … n successfully. Consequently, the class of stage performers is formed by the intersection of all the classes of individual task performers:

S = T_1_ ∩ T_2_ ∩…∩ T_n_.

Ideally, a child must be able to perform all of the tasks in order to be predicated with being at a particular stage; however, it is desirable in the attribution of a stage to avoid bias and hegemony on the one hand and giving excessive weight to a single failure in the face of substantial cognitive achievement on the other. Whilst the dilemma is unavoidable in terms of the dichotomous categories of crisp classes, fuzzy classes appear to offer a way out.

Let µ_S_(c) denote the membership value of a child c in the class of stage performers S. Clearly, the membership value in S will depend on the membership values in T_i_ for i = 1…n just as membership in S is dependent on membership in T_i_’s for crisp classes. Using the rule Zadeh defined for the intersection of fuzzy sets, the membership value in S can therefore be calculated from the membership values of the classes of task performers T_i_ for i = 1…n as follows:

µ_S_(c) = µ_T1 ∩ T2 ∩…∩ Tn_ (c) = min (µ_T1_(c) … µ_Tn_(c)).

For any child, c, then, the membership value in the class of stage performers S would depend on the membership values in the individual classes of task performers.

With this fuzzification, the stage membership value would reflect quantitative variation due to decalage, cultural, socioeconomic, situative, psychological, biological, etc. factors since µ_S_(c) depends on those of the µ_Ti_’s, which are determined on the basis of developmental curves. On the other hand, assessing whether children belong to a stage would not require arbitrary choices between performance in either spatial or conservation tasks for the concrete-operational stage, for example, which would unfairly prejudice against either Baoulé or Inuit children at particular ages, since the criterion for assessment is fixed by the minimum membership value of class performers.

## Conclusions

Genetic epistemology as conceived by Jean Piaget is a science of the growth of knowledge, and, in Piaget’s research programme, the stage theory played a pivotal role. Intelligence was found to develop discontinuously in an invariant sequence of qualitatively discrete steps; stages thus represented a natural classification of cognitive development for Piaget. However, he never regarded the stages as an end in themselves; they were a means to determining the mechanisms of cognitive growth. In other words, the stage theory was a stepping stone in the genetic-epistemological research programme, albeit a necessary one. Nevertheless, it has borne the brunt of much criticism. Broadly, I have touched on some of the criticism commonly levelled at Piaget’s stage theory in this paper, but criticism concerning the lack of discontinuity between Piaget’s stages has taken centre stage.

I have highlighted the compatibility of Piaget’s stage theory with the fuzzy-structuralist model conceived by Seising to represent the continuum connecting theory and empirical research. Stages in the theoretical layer are qualitative, each stage being characterised by specific cognitive structures, and an invariant sequence of stages resulting from qualitative transformations of these structures represents cognitive development. The stage theory is monolithic and universal; however, it pertains to an epistemic subject. In the empirical layer, on the other hand, stages classify the actual performance of children at particular tasks according to success. Theoretization goes hand-in-hand with abstraction from the particulars of the task at hand and the individuals performing them. In addition, it is accompanied by a shift from quantitative change to qualitative transformations. Empirization, on the other hand, goes hand-in-hand with quantification, and it is accompanied by quantitative variation in the rate of progression through the stages measured chronologically by age.

A number of different stage models can be found in Piaget’s work. Some can be disregarded since they belong to an earlier, superseded phase of his work. These notwithstanding, several models were still in use post 1936. In light of fuzzy structuralism, however, one inconsistency in the stage models is understandable. The disparity between empirical stages, which focus on successful performance of specific tasks, and theoretical stages, which emphasise qualitative structures and their transformations, is symptomatic of differences inherent in theoretical and empirical layers of any science. Nevertheless, differences in the stage models within the theoretical layer still remain, leaving Piaget’s stage theory vulnerable to criticism on grounds of inconsistency.

However, the term ‘stage’ is not only employed in purely theoretical or empirical contexts. It is also used in order to characterise the level of cognitive development achieved by a child or groups of children on the basis of empirical evidence. Used in this way, it is not related directly to a theoretical entity, the epistemic subject, nor to the classification of specific performances of a given child at particular tasks according to success; in the words of Hertz (1956, 1; see also Seising 2009, 4), it is one of our ‘conceptions of things’. According to the fuzzy-structuralist model, theory and empirical research permeate each other in the fuzzy space between the two layers. Since stages of cognitive development according to this usage are neither entirely empirical nor entirely theoretical concepts, they appear to inhabit the fuzzy space between the layers, and I have argued that the term ‘stage’ when used in such contexts denotes a fuzzy class. In addition, I have proposed a possible operationalisation of stages in fuzzy space in terms of fuzzy classes. For a given sample group of children, the developmental curves show the proportion of children in any limited age group able to perform a task successfully. Although the curves actually show the proportion of successful performers as a function of age, they could serve as measures of the degree of success as a function of a child’s age, which would allow class membership values for task performers to be derived from these measures. By combining the fuzzy classes of task performers thus derived for each of the tasks designed to assess achievement of the cognitive structures characterising any stage, an operational fuzzification of stages could then be effected.

The stages of cognitive development are clearly characterised in theory, yet there is a great deal of variability in empirical results. Moreover, the qualitative aspects of the stage theory had been largely confirmed experimentally (cf. Saxe, 1981), and variability was thought to be confined to quantitative results alone. In this paper, I have argued that quantification and therefore variability goes hand-in-hand with empirization, but this is only part of the whole picture. According to genetic epistemology, there are theoretical links between the cognitive structures characterising stages both in their final forms and their development. For example, conservation is fundamental to the formation of logical and infralogical structures, although both kinds of structures develop largely independently of each other up to the concrete-operational stage. Moreover, Dasen (1984, 429) has shown that there are not only significant numerical correlations between the performance of tasks within these separate domains of cognition but also correlations between the domains, which led him to believe that the construct validity of stages of cognitive development was not as bad as some critics claimed. Experimental results were, thus, believed to reflect to some extent the interrelatedness of cognitive structures at the theoretical level, despite the quantitative variation due to empirization. It, therefore, seems justified to use the term ‘stage’ to denote the interrelated cognitive performances which reflect the cognitive structures characterising theoretical stages. However, quantitative variation also needs to be acknowledged in its use. Although qualitative changes are the catalystic, real transitions from one stage to the next are not marked by abrupt changes in cognitive capacities.

Cognitive structures characterise the stages of cognitive development in Piaget’s stage theory. In theory, stages thus have crisp contours, and transitions from one stage to the next is discontinuous; however, the crisp contours of the qualitatively discontinuous theoretical stages soften into silhouettes in the fog of empirical variability. The difference between theoretical and empirical stages is reminiscent of the very disparity between theory and empirical research in the sciences that inspired Zadeh to develop fuzzy-set theory, and, focusing especially on the way the concept ‘stage’ is typically used between theory and empirical research, I have argued that a fuzzification of this concept could reconcile the discontinuity of theoretical stages with the gradual blurring of boundaries as quantifiable stages of empirical research gradually replace their qualitatively crisp counterparts. In short, the fuzzy conception of stages as set out in this paper appears to correspond to the way the concept is typically employed; it also appears to do justice to the construct validity on the one hand and quantitative variability in empirical research on the other whilst reconciling the lack of discontinuity in empirical stages compared with their theoretical counterparts.

Finally, the Crisis of Variability was precipitated by quantitative variation in the chronological age of children achieving stages and the rate of progress through them, which did not correspond well with the theoretical expectation of discontinuity between the different stages. Historically, two reactions were evident: some researchers abandoned Piaget’s stage theory for less universal, less monolithic positions, whilst others remained loyal but had to explain away the variability in terms of methodological artefacts (see Dasen and Heron 2000, 92–3). In the light of fuzzy structuralism, the Crisis of Variability can be regarded as symptomatic of the relationship between scientific theory and empirical research. Moreover, fuzzy structuralism, by reconciling theoretical unity and quantitative diversity, shows that there would have been a viable alternative to desertion and denial. Regardless of the proposed conception of stages in terms of fuzzy classes set out in this paper, then, the alternative suggested by fuzzy structuralism indicates that the theoretical unity and empirical variability in research on cognitive development are not necessarily irreconcilable; in fact, they can represent two sides of the same coin even when one side is qualitative and the other quantitative.

## Data Availability

Not applicable

## Footnotes

1 According to Inhelder (1989, ix), Piaget first systematically addressed the role of the object in cognition in the third phase of his work, which dealt with the ontogenesis of causality amongst other things.

2 Contrasting the theoretical and empirical aspects of Piaget’s stage theory, Rose and Fischer (2009, 404) write ‘Piaget predicted that as a new logic emerged in the mind, it would catalyze the whole mind into a new kind of intelligence. However, research has consistently found unevenness instead of monolithic transformation, even with logically equivalent tasks’. I use the term ‘catalytic’ in this sense.

3 Brainerd (2000) distinguishes three typical uses of stages in theories of behavioural development: aesthetic, descriptive and explanatory. According to him, the term ‘stage’ is used in the latter two senses in Piagetian theory but not in the first. With regard to the descriptive sense, he draws attention to a descriptive continuum in the use of stages in Piagetian theory reaching from the concrete to the abstract that corresponds well with the continuum in fuzzy space between the empirical and theoretical layers. As for the explanatory sense, he sees evidence for statements to the effect that ‘children do or do not do such and such because they are at some given stage of cognitive development’ (Brainerd, 2000, 66) in Piagetian and non-Piagetian circles. He does not consider such statements to be fully justified; however, the validity of these statements as explanations is not important for the present purposes; what matters is that a stage is being attributed to a child simply on the basis of performance at particular tasks.

4 Dasen and Heron (2000, 90–1) are also interested in the performance of a particular child at a particular task. They propose an extension of a model suggested by Flavell and Wohlwill. Accordingly, the probability that a given child will solve a given task can be formulated as follows: P(+) = P_a_ × P_b_^1−k^ × P_c_^k^, where 0 < P_a_<1; 0 < P_b_<1; 0 < k < 1 ; and P_a_ reflects competence, i.e., the presence or absence of an operational structure. An attribute of the task, P_b_ represents the likelihood for any given task that the operation will in fact be called into play, and its end product will be translated into the desired output. The task-related variables vary with age, and age is taken into account by introducing a parameter *k.* In addition to the competence and task-related variables, Dasen and Heron add the variable P_c_ representing the likelihood for any given task that the operation will in fact be called into play in a given cultural milieu. Moreover, they believe that the cultural effects will increase with age, hence P_c_ is raised to the power of *k.* Their model is based on probability, and its goal is prediction of the successful performance of a task for a given child rather than the calculation of a membership value, which would be useful for the application of a concept.

## References

[CR1] Brainerd, C. J. (2000). The Stage Question in Cognitive-Development Theory. In L. Smith (Ed.), *Jean Piaget Critical Assessments*, Reprint 2000, 4:71–86. London; New York; Canada: Routledge

[CR2] Dasen PR (1975). Concrete Operational Development in Three Cultures. Journal of Cross-Cultural Psychology.

[CR3] Dasen PR (1984). The Cross-Cultural Study of Intelligence: Piaget and the Baoulé. International Journal of Psychology.

[CR4] Dasen, P. R. & Heron, A. (2000). Cross-Cultural Tests of Piaget’s Theory. In L. Smith (Ed.), *Jean Piaget Critical Assessments*, Reprint 2000, 3:64–110. London; New York; Canada: Routledge

[CR5] Dasen PR, de Ribaupierre Anik (1987). Neo-Piagetian Theories: Cross-Cultural and Differential Perspectives. International Journal of Psychology.

[CR6] Hertz H (1956). The Principles of Mechanics Presented in a New Form.

[CR7] Inhelder B (1989). Foreword. Psychogenesis and the History of Science.

[CR8] Inhelder, B. (2000). Some Aspects of Piaget’s Genetic Approach to Cognition. In L. Smith (Ed.), *Jean Piaget Critical Assessments*, Reprint 2000, 1:23–39. London; New York; Canada: Routledge

[CR9] Kesselring T, Mueller U, Carpendale JIM, Smith L (2009). The Mind’s Staircase Revised. The Cambridge Companion to Piaget.

[CR10] Lloyd BB (1971). The Intellectual Development of Yoruba Children: A Re-Examination. Journal of Cross-Cultural Psychology.

[CR11] Montangero J (2000). The Various Aspects of Horizontal Décalage. Jean Piaget Critical Assessments.

[CR12] O’Brien DP, Overton Willis F. (1982). Conditional Reasoning and the Competence-Performance Issue: A Developmental Analysis of a Training Task. Journal of Experimental Child Psychology.

[CR13] Overton W, Newman Judith, Field TM, Huston A, Quay HC, Troll L, Finley GE (1982). Cognitive Development: A Competence-Activation/Utilization Approach. Review of Human Development.

[CR14] Piaget, J. (1970). *Structuralism*. Translated by C. Maschler. New York: Basic Books Inc

[CR15] Piaget, J. (1977). The Stages of Intellectual Development in Childhood and Adolescence. In H. E. Gruber and J. -J. Vonèche (Eds.), *The Essential Piaget*, 814–819. New York: Basic Books Inc

[CR16] Piaget, J. (1985). *The Equilibration of Cognitive Structures: the Central Problem of Intellectual Development*. Translated by T. Brown and K. J. Thampy. Chicago: University of Chicago Press

[CR17] Piaget, J. (2001). *The Psychology of Intelligence*. Translated by M. Piercy and D. E. Berlyne. Routledge Classics. London; New York: Routledge

[CR18] Piaget, J. & Garcia, R. (1991). *Toward A Logic of Meanings*. P. Davidson and J. Easley (Eds.). Hove, London: Lawrence Erlbaum Associates, Inc

[CR19] Piaget, J., & Grize, J.-B. (1972). *Essai de logique opératoire* (2e éd. *du Traité de logique, essai de logistique opératoire* (1949)., Vol. 15). Paris: Dunod.

[CR20] Rosch E (1973). Natural Categories. Cognitive Psychology.

[CR21] Rose, L., Todd, & Fischer, K. W. (2009). Dynamic Development: A Neo-Piagetian Approach. In U. Mueller, J. I. M. Carpendale, & L. Smith (Eds.), *The Cambridge Companion to Piaget* (pp. 400–420). Cambridge University Press

[CR22] Saxe GB (1981). When Fourth can Precede Second: A Developmental Analysis of an Indigenous Numeration System among Ponam Islanders in Papua New Guinea. Journal of Cross-Cultural Psychology.

[CR23] Seising, R. (2007). *The Fuzzification of Systems. The Genesis of Fuzzy Set Theory and Its Initial Applications. Its Development to the 1970s*. Studies in Fuzziness and Soft Computing 216. Berlin: Springer

[CR24] Seising R, Seising R (2009). Fuzzy Sets and Systems and Philosophy of Science. Views on Fuzzy Sets and Systems.

[CR25] Seising, R. (2013). A “Goodbye to the Aristotelian Weltanschauung’” and a Handbook of Analytical Philosophy of Medicine. In R. Seising and M. Tabacchi (Eds.), *Fuzziness and Medicine: Philosophical Reflections and Application Systems in Health Care. A Companion Volume to Sadegh-Zadeh’s “Handbook on Analytical Philosophy of Medicine”*. Studies in Fuzziness and Soft Computing. Berlin, New York: Springer

[CR26] Zadeh, L. A. (1962). From Circuit Theory to System Theory. In *Proceedings of the IRE*, 50:856–865

[CR27] Zadeh LA (1965). Fuzzy Sets. Information and Control.

[CR28] Zadeh LA, Fox J (1965). Fuzzy Sets and Systems. System Theory.

